# Probiotics’ Effects in the Treatment of Anxiety and Depression: A Comprehensive Review of 2014–2023 Clinical Trials

**DOI:** 10.3390/microorganisms12020411

**Published:** 2024-02-19

**Authors:** Ermis Merkouris, Theodora Mavroudi, Daniil Miliotas, Dimitrios Tsiptsios, Aspasia Serdari, Foteini Christidi, Triantafyllos K. Doskas, Christoph Mueller, Konstantinos Tsamakis

**Affiliations:** 1 Neurology Department, Democritus University of Thrace, 68100 Alexandroupoli, Greece; ermimerk@med.duth.gr (E.M.); theodoramavroudi148@gmail.com (T.M.); miliotasdanos@gmail.com (D.M.); tsiptsios.dimitrios@yahoo.gr (D.T.); christidi.f.a@gmail.com (F.C.); 2 3rd Neurology Department, Aristotle University, 54124 Thessaloniki, Greece; 3 Department of Child and Adolescent Psychiatry, Medical School, Democritus University of Thrace, 68100 Alexandroupolis, Greece; aserntar@med.duth.gr; 4 Neurology Department, Athens Naval Hospital, 11521 Athens, Greece; doskastr@gmail.com; 5 Institute of Psychiatry, Psychology and Neuroscience (IoPPN), King’s College London, London SE5 8AB, UK; christoph.mueller@kcl.ac.uk; 6 Biomedical Research Centre, South London and Maudsley NHS Foundation Trust, London SE5 8AF, UK; 7 Institute of Medical and Biomedical Education, St George’s, University of London, London SW17 0RE, UK

**Keywords:** probiotics, anxiety, depression, microbiome, gut, microbiota, alterations, review, prebiotics, psychobiotics

## Abstract

Changes in the gut microbiome can affect cognitive and psychological functions via the microbiota–gut–brain (MGB) axis. Probiotic supplements are thought to have largely positive effects on mental health when taken in sufficient amounts; however, despite extensive research having been conducted, there is a lack of consistent findings on the effects of probiotics on anxiety and depression and the associated microbiome alterations. The aim of our study is to systematically review the most recent literature of the last 10 years in order to clarify whether probiotics could actually improve depression and anxiety symptoms. Our results indicate that the majority of the most recent literature suggests a beneficial role of probiotics in the treatment of depression and anxiety, despite the existence of a substantial number of less positive findings. Given probiotics’ potential to offer novel, personalized treatment options for mood disorders, further, better targeted research in psychiatric populations is needed to address concerns about the exact mechanisms of probiotics, dosing, timing of treatment, and possible differences in outcomes depending on the severity of anxiety and depression.

## 1. Introduction

Several microbial environments exist in different locations inside the human body, such as the skin, nasal mucosa, and gastrointestinal tract, consisting of a large number of bacteria, viruses, fungi, and protozoa, referred to as the microbiota [1]. Thousands of different microbe species have been identified in human beings, with most of them classified in the *Proteobacteria*, *Firmicutes*, *Actinobacteria*, and *Bacteroidetes* phyla [2]. The microbiome (i.e., the entire environment, including the microorganisms, their genomes, and the surrounding environmental conditions) [3] of the gut contains the largest and most diverse population of microorganisms, that are critical for a variety of physiological functions, including preserving gut integrity or forming the intestinal epithelium [4], obtaining energy [5], protecting against infections [6], and controlling host immunity [7]; it is thus considered an “invisible organ” of the human body [8].

The gut–brain axis is a system which encompasses a bidirectional communication between the gut and the brain [9] involving the neural (vagus nerve and enteric nervous system), immune (cytokines), endocrine (cortisol), and metabolic (short-chain fatty acids) pathways [10,11]. Gut-produced cytokines can reach the brain through the bloodstream and although it is doubtful that they would cross the blood–brain barrier (BBB) under normal physiological conditions, there is growing evidence that they can affect parts of the brain where the BBB is inadequate, such as the hypothalamus; for instance, cytokines interleukin-1 (IL-1) and IL-6 trigger the release of cortisol by activating the hypothalamic–pituitary–adrenal (HPA) axis [12]. In addition, gut microbiota is the primary source of short-chain fatty acids (SCFAs), which modulate brain health and behavior via the immune system, having a beneficial anti-inflammatory and mental health role by inducing T cell differentiation, controlling inflammatory cytokine production, and influencing serotonin and other neurotransmitter production [13]. The gut microbiome is also able to metabolize glutamate to produce certain beneficial metabolites, such as GABA and serotonin, which are known molecules thought to reduce anxious and depressive states [14]; despite not being able to cross the blood–brain barrier (BBB), they can nevertheless pass through the intestinal mucosal layer and have an indirect effect on brain function via the enteric nervous system [15]. As a result, the gut microbiome plays a central role in modulating the reciprocal communication between the gut and brain and together they comprise the microbiota–gut–brain (MGB) axis [3]. Increasing evidence suggests that the gut microbiome and the MGB axis play a crucial role in modulating psychiatric disorders [16]. Therefore, treatments such as probiotics that potentially alter the composition and function of the gut microbiome [17], might have desired beneficial effects in human mental health [18].

Probiotics are described as “living microorganisms” that when administered in adequate amounts confer health benefits on the host [19]; psychobiotics are defined as those probiotics that can specifically bestow mental health benefits. This idea has been extended to include “prebiotics”, that promote the proliferation of beneficial bacteria in the gastrointestinal tract [20]. The combination of probiotics and prebiotics is often described as synbiotics; however, the term has been recently redefined as “a mixture comprising live microorganisms and substrate(s) selectively utilized by host microorganisms that confers a health benefit on the host” [21]. Existing evidence suggests that probiotics may help patients with conditions such as IBS and persistent exhaustion feel emotionally better and live with less stress [22], while certain elements of brain function and behavior, particularly those that are vagus nerve dependent, can be altered by probiotic strains [23]. The mechanisms of action of probiotics are diverse, heterogeneous, and strain specific [24]. The beneficial effects of probiotics have largely been attributed to the modification of the intestinal microbiota, through improving the functioning of the microbial populations already present and thus limiting pathogens [25]. It is also accomplished through the colonization and normalization of disrupted intestinal microbial communities in humans, alongside the competitive exclusion of pathogens and the production of bacteriocins [24]. Moreover, probiotics have been found to immunomodulate the body, promote the growth and differentiation of epithelial cells, reinforce the intestinal barrier [26], and create antimicrobial substances or metabolic products that inhibit the growth of other possibly harmful bacteria [17], while also fighting with them for receptors and binding points on the intestinal mucosa [27], preserving the gut microbiome’s harmony [28].

The effect of probiotics in anxiety and depression is currently being extensively investigated given their potential to be novel, adverse event-free, and customizable treatments [18]. Clinical trials in humans have overall shown that probiotic and prebiotic therapies have beneficial mental health effects. For instance, the probiotic combination of *L. helveticus* and *B. longum*, when given to healthy human volunteers for one month, has been shown to reduce psychological distress [29], while patients with IBS who received high intake of prebiotic *trans-GOS* for 12 weeks reported significantly improved subjective global assessment scores and anxiety scores compared to placebo treatment [30]. Moreover, participants in a healthy state who consumed a milk drink containing probiotics on a daily basis presented with a considerable boost in their mood after three weeks when compared to a placebo [31].

When looking at meta-analytic data, two relatively older studies from 2016 and 2017 examining the effectiveness of probiotics in alleviating depression revealed a general improvement of depressive symptoms following probiotic ingestion, with minimal potential for negative side effects [32,33]. A more recent meta-analysis supported the use of probiotics in depressed patients with or without concomitant somatic disorders; however, it could not draw firm conclusions on the effectiveness of probiotics in the healthy population [34]. On the contrary, another meta-analysis showed that probiotics can reduce subjective stress level in healthy volunteers [35]. A 2021 meta-analysis demonstrated with moderate- and low-certainty evidence that probiotics improve symptoms of depression and anxiety in clinical patients measured with the Beck Depression Index and the State–Trait Anxiety Inventory but not on any other scales [36].

However, other meta-analytic data have been less positive. A recent study demonstrated that most probiotics did not affect mood, stress, anxiety, depression, and psychiatric distress when compared to placebo at the qualitative level, indicating that there is not yet strong enough evidence to support the inclusion of probiotic, prebiotic and symbiotic supplements in treatment guidelines for depression [37]. Another meta-analysis suggested that the current evidence base for prebiotics and probiotics in the treatment of internalizing disorders appears modest [38], while a 2020 meta-analysis reported that not all probiotic treatments exhibited a psychobiotic effect on the central nervous system (CNS) [39]. Moreover, another study found no significant difference between the probiotics and placebo groups in alleviating anxiety and suggested that probiotics should be used in clinical subjects or the general population only when more valid evidence in this area is obtained [40].

Therefore, despite the vast scale of research that has been taking place in the multibillion dollar probiotic industry [18] in the last years there is a lack of consensus of the existing evidence on how to best use them in mental health, which might cause confusion in healthcare professionals [41]. Thus, and given the increasingly broader range of probiotic products used [41] and the rapidly increasing literature in the probiotic field [42], the aim of our study was to provide a comprehensive update of the last decade research data on the effectiveness of probiotics in the treatment of anxiety and depression. Taking into consideration the huge potential of probiotics to become novel, personalized treatments for mental health disorders, the academic importance of our study lies in the effort to analyze the most recent (last ten years) accumulated knowledge in the field and shed more light on the question of whether and to what degree probiotics are actually effective in depression and anxiety treatment.

## 2. Materials and Methods

The Preferred Reporting Items for Systematic Reviews and Meta-Analyses (PRISMA) checklist was used to guide this study. A protocol has not been registered; however, our study methods were designed and formulated a priori.

### 2.1. Search Strategy

Two databases (MEDLINE and Scopus) were selected to carry out the present literature search, which was conducted by two investigators (TM, DM). To trace all relevant studies published between 1 January 2014 and 31 December 2023, the following keywords were used: “probiotics” OR “prebiotics” OR “psychobiotics” AND “gut microbiota” OR “gut microbiome” AND “depression” OR “anxiety”. All retrieved articles were also hand searched for any further potential eligible articles. Any disagreement regarding the screening or selection process was solved by a third investigator (EM) until a consensus was reached.

### 2.2. Selection Criteria

Only full-text original research articles (randomized controlled trials and clinical trials) published in the English language were included. Secondary analyses, reviews, guidelines, notes, errata, letters, meeting summaries, comments, unpublished abstracts, retracted articles, or studies conducted on animals or children (<18 years old) were excluded. There was no restriction on study design or other sample characteristics.

### 2.3. Data Extraction

Data extraction was performed independently by two investigators from the team (DT and KT) using a predefined data form created in Excel. The same two investigators also rated the quality of the included studies using the Jadad scale [43], also known as the Oxford quality scoring system, in order to evaluate the methodological quality of clinical trials and subsequently assess the bias of the included studies. The Jadad scale consists of three items: (A) randomization (0–2 points), (B) blinding (0–2 points), and (C) study dropouts and withdrawals (0–1 points). For each item, there is a “yes” or “no” answer option, which results in 1 or 0 points, respectively. Studies can be given a score between 0 and 5, with a score of <3 indicating that the study is of poor quality, while studies are classified as high quality if they have a Jadad score of 3 or more. The Jadad scale is a well-known and well-applicable instrument with good reliability and validity evidence [44].

We recorded the authors, year of publication, the type of study, type or types of questionnaires for anxiety, stress, or depression assessment, the strains of microorganisms contained within the probiotic supplement, and finally, the main findings of each study. Possible discrepancies during data extraction were solved via discussion with a third investigator (EM).

### 2.4. Data Analysis

No statistical analysis or meta-analysis was performed due to the high heterogeneity among the studies. Thus, the data were only descriptively analyzed.

## 3. Results

### 3.1. Database Searches

**Figure 1:**
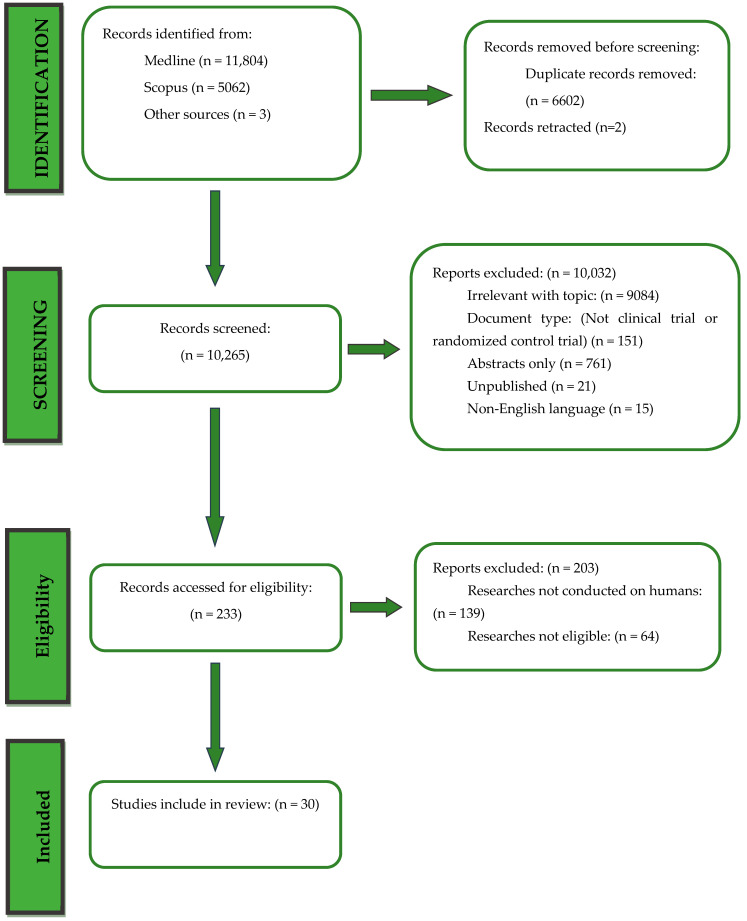
Flowchart of studies’ selection.

Overall, 16,869 records were retrieved from the database search. Duplicates were removed; hence, a total of 10,265 articles were selected. After dismissing irrelevant studies and screening the full texts of the articles, 30 studies were eligible for inclusion (Figure 1).

### 3.2. Studies Characteristics

Thirty publications fulfilled our inclusion criteria. Four studies focused entirely on depression, and four reported only on anxiety or stress, while twenty-two assessed both anxiety and depression scores. Concerning the type of the studies, twenty-eight were randomized controlled trials and only two were clinical trials.

The vast majority of the studies (83.3%) were of high quality. The quality scores of the included studies are summarized in Supplementary Table S1.

### 3.3. Microorganism Strains

Twenty-five studies utilized *Lactobacillus* and nineteen utilized *Bifidobacterium* genera, while others used a variety of microorganism genera, such as *Clostridium (2)*, *Lactococcus (3)*, *Actobacillus (1)*, *Streptococcus (2)*, and *Lactiplantibacillus (1).* Most of them utilized a combination of some of the aforementioned probiotics. The most used strains were as follows: *Bifidobacterium longum* (twelve studies), *Bifidobacterium lactis* (eleven studies), *Lactobacillus acidophilus* (ten studies), *Lactobacillus casei* (eight studies), and *Lactobacillus helveticus* (seven studies). The summary of all the strains used in each probiotic supplement can be seen on Table 1.

### 3.4. Assessment Tools for Anxiety and Depression

There was a variety of different kinds of assessment tools used for evaluation of anxiety and depression. The most frequently used ones were the Beck Depression Inventory (BDI) (nine studies), Depression, Anxiety, and Stress Scale (DASS) (eight studies), State–trait Anxiety Inventory (STAI), and Perceived Stress Scale (PSS) (seven studies), while Hospital Anxiety and Depression Scale (HADS) was utilized in six studies. In addition, other often used questionnaires included Hamilton Rating Scale of Depression (HAMD)- five studies, Beck Anxiety Inventory (BAI) (five studies), and the Hamilton Anxiety Rating Scale (HAMA) (four studies). Apart from that, there was also a number of 30 additional scales used, which can be seen on Table 1.

### 3.5. Treatment Duration

The most frequent strategy of treatment length was around 8 weeks, as was followed in eleven studies. Also, frequent treatment length was the 4-week (seven studies) and 2-week (five studies) duration. Additionally, three studies were conducted on pregnant women, thus the treatment length was equivalent to the weeks of pregnancy, while in one of these studies the treatment continued for another 6 months whilst breastfeeding. The summarized results can be seen in Table 1.

### 3.6. Clinical Outcomes

Most of the studies included in this review support the beneficial effects of probiotics. Kazemi et al. [46] demonstrated a significant decrease in BDI score (17.39–9.1) compared to placebo (18.18–15.55) (*p* = 0.042), after an 8-week use of a probiotic supplement as the main treatment, while Gawlik-Kotelnicka et al. also demonstrated clinically significant improvement in MADRS and QoL (quality of life) scores [63]. On the same basis, Schaub et al. [45] showed significant improvement in depression at the 8-week follow up, with a remission rate of 55% in the probiotics group compared to a 40% in the placebo group, but only for the one subgroup with high compliance of a short-term, high-dose intake of probiotics as a supplementary treatment for depression. Regardless of the type of antidepressant used, Miyaoka et al. discovered that taking *Clostridium butyricum MIYAIRI 588* in treatment-resistant depression (TRD) led to a reduction in various depression and anxiety scores by more than 50% at the end of the 8-week study [60]. On the same note, Chen et al.’s study [54] supported the benefits of probiotics at 8 weeks, while Ho et al.’s [61] and Lee et al.’s [47] studies showed that the probiotic groups presented with fewer depressive symptoms and improvements in sleep. More specifically, in Lee’s study those taking the NVP-1704 exhibited a greater decline in the BDI-II score compared to those on the placebo, at both the third (−6.18 ± 7.34 vs. −3.33 ± 7.03, *p* = 0.033) and final visits (−8.02 ± 7.17 vs. −5.39 ± 6.49, *p* = 0.036). Moreover, Yang et al.’s study [51] suggested that probiotic therapy led to reduced psychological stress before surgery, preventing patients’ anxiety and heart rate from increasing. Similarly, Nikolova et al. [71] demonstrated a reduction in depressive symptoms in patients in the probiotic group with modest symptoms (HAMD-17 scores week 4: standardized effect sizes (SES), 0.70; 95% CI, 0.01–0.98, and IDS Self Report scores week 8: SES, 0.64; 95% CI, 0.03–0.87), with probiotic consumption being particularly helpful in reducing anxiety-related somatic symptoms (HAMA scores week 4: SES, 0.67; 95% CI, 0–0.95; week 8: SES, 0.79; 95% CI, 0.06–1.05). In another study, multiple sclerosis (MS) patients with depressive symptoms taking probiotic capsules for 12 weeks had significantly decreased EDSS, BDI, GHQ, and DASS scores [75], while Pinto-Sanchez et al.’s study [50] used patients with IBS and found a reduction in depression levels, but not in anxiety, when the probiotic *Bifidobacterium longum NCC3001* was administered; the improvement in HAD-D scores was sustained at the 10-week follow up.

Adikari et al. [74] used EEG to investigate the effects of probiotics on physiological parameters related to anxiety in football players; after 4 weeks, they found higher delta and theta brain waves in the probiotic group, indicating, mainly that the patients were more relaxed, and their alertness was increased. Raygan et al. [70] investigated the effect of simultaneous treatment with vitamins and probiotics and showed that the intake of probiotics and vitamin D in patients with T2DM and congestive heart failure over a period of 12 weeks had positive effects on mental health parameters, including stress and depression.

Two studies utilized students as a patient group. In a study by Zhu et al. [53], the probiotic supplement prevented worsening of anxiety and significantly improved depression and sleep quality in students with mild anxiety and depression, as was also reported in another study by Venkataraman et al. [73] in students with moderate anxiety. In contrast Tran et al. [55] suggested that the beneficial effect is present only in people with severe anxiety. Similarly, Boehme et al. [66] suggested that supplementation with *BL NCC3001* can improve the perception of stress. Two other studies looked at synbiotics as a treatment: Hadi et al. [72], showed that consumption of the supplement for eight weeks led to a significant improvement in stress (−3.49 ± 2.30 vs. −1.41 ± 3.44; *p* < 0.001), anxiety (−2.61 ± 1.49 vs. −1.46 ± 2.49; *p* = 0.03), depression (−2.86 ± 2.47 vs. −1.54 ± 2.13; *p* = 0.03) when compared to placebo. Similarly, Haghighat et al. [62] demonstrated the beneficial effects of synbiotics in depression (mean decrease in HADS-DEP score = 1.28, *p* = 0.009).

On the contrary, there were several studies that found subtle to no effects of probiotics on anxiety or depression. In a study by Lew et al. [68] the probiotic strain *L. plantarum P8* was used in people with moderate stress, and even after 12 weeks there was no significant improvement in depression symptoms. Similarly, in Rode et al.’s study [49], although the probiotics intervention tended to lower HADS scores compared to placebo, none of the questionnaires or the Actigraphy stress ratings were associated with significant improvement due to the intervention. On the same note, Östlund-Lagerström et al. [67] and Rudzki et al. [57] also reported that the patients’ symptoms did not significantly improve during the course of the treatment. Romijin et al. [58] suggested that the lack of effect of probiotics on psychological outcomes in their study was due to the length of the intervention period (8 weeks), sample size or the fact that there was no other treatments used other than probiotics. Finally, Morales-Torres et al. [59] found no significant effects and concluded that lifestyle behaviors play a role not only in mental health but also in the efficacy of probiotics; in fact, the interaction between a “good” lifestyle and probiotic intake was the only significant predictor of positive effects on anxiety, emotion regulation difficulties and mindfulness in post-treatment outcomes when controlling for gender and age.

Interestingly, in two studies, the improvement in depressive and anxiety symptoms were statistically important in both the treatment and the placebo groups [48,52]. In Reininghaus’ study [52] where biotin (B7) was used in combination with a multi-strain probiotic in addition to the standard treatment of depressed patients, although psychiatric symptoms improved over time in both groups, there were no differences in the outcome measures between the treatment and the placebo groups that. On the same basis, in Chahwan’s study investigating the efficacy of the multispecies probiotic Ecologic Barrier in the treatment of depression, participants in both the probiotic and placebo groups showed a reduction in depressive symptoms. Analyses of one-month follow-up data revealed no significant between group differences on BDI, BAI, cognitive reactivity, and DASS scores (*p* > 0.05). Even after dividing the participants into groups according to severity of symptoms (mild/moderate and severe), the probiotic group did not show a greater degree of reduction in depressive symptoms. The only notable difference was found in the mild/moderate depression severity group on a test of cognitive reactivity to depressive mood, a psychological vulnerability marker suggesting that the beneficial effects of probiotics may be more noticeable in people with milder depression.

**Figure 2:**
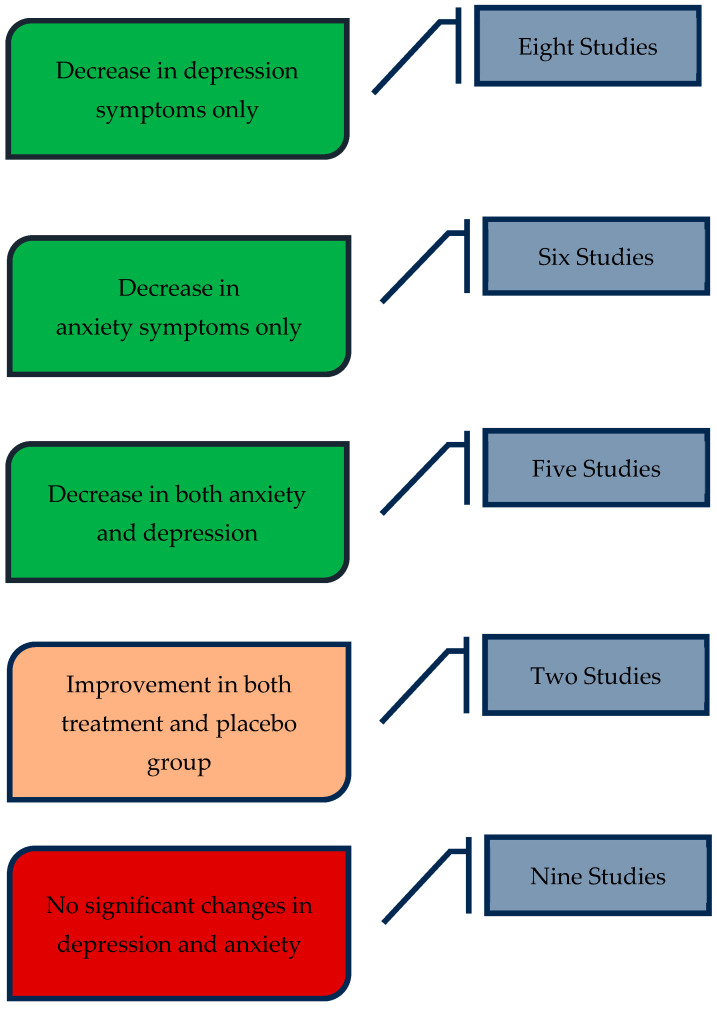
Clinical outcomes of probiotic use in anxiety and depression.

A summary of the findings in clinical outcomes can be seen in Figure 2.

### 3.7. Maternal Health

Three studies concentrated on maternal depression and anxiety [56,64,65] with conflicting results. In Slykerman et al.’s study [56], pregnant women taking the probiotic *Lactobacillus rhamnosusHN001* had fewer signs of postnatal anxiety and depression, compared to placebo (depression scores HN001 mean = 7.7 (SD = 5.4), placebo 9.0 (6.0), effect size −1.2, (95% CI −2.4, −0.1), *p* = 0.035), anxiety scores (HN001 12.0 (4.0), placebo 13.0 (4.3), effect size −1.1 −1.9, −0.2), *p* = 0.014). In addition, the study followed up with the women after birth and showed an association between higher levels of anxiety and depression and infant colic, which indicated that probiotic supplements could improve the mother’s mood by relieving the infant’s colic. After controlling for infant colic and time since birth, the reduction in anxiety (*p* = 0.014) and depression (*p* = 0.037) maintained in the long term, i.e., in the few months after birth. However, in the study by Browne et al. [65], pregnant women experienced no change in psychosocial distress after 8 weeks of probiotic consumption, and there were no differences in depressive symptomatology between the probiotic and placebo group in the 4-week postpartum follow up either. Similarly, Dawe et al. [64] found no changes in depression or anxiety in pregnant obese women between the prenatal baseline and the end of the study at week 36.

### 3.8. Microbiota Diversity and Abundance

Alterations in the microbiome diversity and certain genera abundance were investigated in a number of the included studies. Lee et al. [47] suggested that while the probiotic *NVP-1704* showed very subtle increases in alpha and beta diversity, it induced an increase in *Bifidobacterium* and *Lactobacillus* strains and a decrease in *Proteobacteria*. In Schaub et al.’s study [45], the probiotics maintained microbial diversity and richness and instigated an increase in the abundance of the genus *Lactobacillus* after the intervention. On the same basis, Haghighat et al. [62] reported that in the synbiotic and probiotic supplemented groups, the fecal *Bifidobacteria* and *Lactobacilicolonies* numbers increased, and the coliform colonies number decreased at week 12 in comparison to the baseline. Chen et al. [54] reported that the number of genus *Akkermansia* increased in major depressive disorder patients after taking probiotics, while Reininghaus et al. [52], although they found no alterations in alpha diversity, demonstrated significantly altered beta diversity and a significant increase in global differential abundance of *R. gauvreauii* and the related *Coprococcus 3*. Similarly, Zhu et al. [53] demonstrated that with the use of *L. plantarum*, the beta diversity of the gut microbiota of patients taking probiotics was somewhat different from that of anxious students taking the placebo.

On the contrary, several studies revealed no significant alterations in the microbiome composition. In Rode et al.’s study [49], it was found that a 4-week probiotic product supplement did not alter the composition of the microbiota. Similarly, Chen et al. [54] indicated that the consumption of *L. plantarum PS128* did not induce any significant changes in the microbiome. Similarly, there were no significant differences in abundance of bacterial taxa or a-b diversities in Chahwan’s study [48]. Meanwhile, by examining fecal 16S rRNA gene sequencing, Pinto-Sanchez et al. [50] found that the beneficial effect of *BL* did not depend on significant changes in microbial abundances or diversity.

### 3.9. Inflammation Biomarkers

Several studies measured inflammation biomarkers (TNF-a, IFNγ, IL-6) or molecules of the ACTH-cortisol signaling pathway. Venkataraman et al. [73] demonstrated that probiotics significantly reduced serum cortisol, which is associated with stress and depression, possibly by dysregulating gut barrier function and indirectly stimulating inflammatory immune responses. Although the cortisol fluctuations were very small, Lew et al. [68] found reduced levels of pro-inflammatory cytokines such as TNF-a and IFNγ in plasma, after treating with probiotics, suggesting that inflammation may have contributed to stress and anxiety. Moreover, according to the findings of Lee et al. [47], *NVP-1704* lowered serum IL-6 levels and alleviated depression and anxiety attributed to the control of certain microorganisms via neuroinflammatory signaling pathways.

Chen et al. [54] also reported correlations between fluctuations in IL-6 levels and the abundance of the genus *Akkermansia*. Additionally, Gawlik-Kotelnicka et al. [63] reported reduced inflammation marker levels due to probiotics usage, while Miyaoka et al. [60] also suggested that *C. butyricum* M588 may have significant anti-inflammatory effects. Furthermore, recent data from Yang et al. [51] showed that serum corticotrophin-releasing hormone (CRF) levels and heart rate of healthy controls remained unchanged after taking probiotics suggesting that taking probiotics has no effect on the body’s physiological CRF.

However, markers of the hypothalamic–pituitary–adrenal axis, including serum levels of kynurenine, tryptophan, neuropeptide Y, and ACTH-cortisol signaling pathway, were not affected by the probiotics in two studies [47,73] and four studies [49,50,57,58] demonstrated that probiotic usage did not affect any inflammation biomarker or cortisol in the subjects in neither of the groups. Finally, Haghighat et al. [62] also reported a statistically significant difference in serum brain-derived neurotrophic factor (BDNF) in the symbiotic but not in the probiotic group, while Rode et al. [49] suggests that BDNF and serotonin increased subtly, but not significantly.

### 3.10. Changes in Metabolic Profile

Several of the included studies measured changes in certain metabolites related to the gut microbiome, which is influenced by the probiotic usage, and their association to depression and anxiety. Rudzki et al. [57] found a significant decrease in kynurenine with the use of *Lactobacillus Plantarum 299v*, which was accompanied by an improved cognitive performance. In addition, Rode et al. [49] suggested that probiotics had subtle effects on markers of gut–brain interaction, such as serum serotonin concentrations, while other studies also suggested that probiotics act through serotonin pathways [72].

Moreover, Pinto-Sanchez et al.’s [50] study showed that *B. Longum* altered the urine metabolic profile, with methylamines and aromatic amino acids being degraded to a lesser extent by bacteria; increased 4-cresol sulfate levels were associated with lower depression scores in the *B. Longum* group. In addition, Zhu et al.’s study [53] showed that the probiotic treatment was linked to 13 fecal metabolomics, nearly all of which showed a negative correlation with the disrupted gut microbiome.

## 4. Discussion

The present review provides an overview of the potential clinical use of probiotics (including prebiotics/psychobiotics/synbiotics) as a therapeutic tool in the treatment of depression and anxiety. Nearly two thirds of the included studies support the modest benefit of a probiotic supplement in depression and/or anxiety (thirteen studies on depression and eleven on anxiety). Moreover, probiotic treatment appeared to be particularly helpful not only in patients with an active psychiatric illness, but also in healthy individuals experiencing stressful life events, which is in line with existing meta-analytic evidence [32]. The vast majority of the studies did not report a long-term follow-up period, whilst very few described a short-term follow up of a few weeks post intervention; although no conclusions could not be drawn, due to limited data, there were no differences identified regarding anxiety/depression scores in the follow-up assessment compared to the end of intervention. It should also be noted that in nearly one third of the included studies (nine studies in total) no significant benefit was noted after the consumption of probiotics, which again is in concordance with other existing data [37,40]. These conflicting findings could partly be attributable to the significant heterogeneity in the strains used in our included studies.

Interestingly, in a couple of studies, although there was improvement in the clinical picture, clinical outcomes were improved in both the treatment and the control group suggesting that preparing and consuming the probiotic daily, making appointments with the hope to improve depressive symptoms had a positive effect on mood, regardless of whether the probiotic or placebo was taken. This, along with the fact that mild depressive episodes are more likely to respond to placebo [76] and the differences noted in the mild/moderate depression severity group in one of the aforementioned studies [48], indicates that individuals experiencing milder depression may exhibit more pronounced positive outcomes from probiotics. In summary, our findings indicate that the majority of the clinical trial evidence from the last decade supports the use of probiotics in (possibly milder) depression; however, at the same time, a substantial amount of evidence shows insufficient results.

The beneficial effect on probiotic use in maternal/pregnancy mental wellbeing was also unclear; the conflicting results could be explained not only due to the small number of studies, but also due to demographic differences, dosage [77], host diet, genetics, age, medication use [78,79], and the severity of the initial symptoms, which could influence the self-reported date, as demonstrated in older studies [31].

The overall evidence in our review on the alterations induced by probiotics in gut microbiota’s diversity and certain genera abundance are limited. Previous studies have shown that MDD and generalized anxiety disorder (GAD) have been associated with an increase in abundance of *Proteobacteria* and *Enterobacteriaceae* [80,81]; this is in line with one of our included studies, where probiotic use was associated with a decrease in *Proteobacteria* [47]. Also, our review indicated that probiotic use can lead to increased abundance of *Lactobacillus* [45,47], species of which, such as *L. casei* [82], *L. rhamnosus* [83], and multi-strain products containing *L. plantarum* [53] have been implicated in modulation of psychiatric disorders and stress-induced behaviors. However, a significant proportion of the studies revealed no alterations the microbiota composition; this could partly be attributed to participants maintaining their usual dietary and lifestyle habits and the intake of the probiotic being limited to a few weeks. Findings on the alterations in alpha and beta diversity were also limited and somewhat contradictory. In summary, we were not able to detect consistent, clear evidence of specific microbiome changes following probiotic consumption.

Nearly half (thirteen) of our included studies suggested that the use of probiotics lead to decreased inflammation markers or potentially harmful metabolic molecules, however their role still needs to be elucidated. For instance, in one study probiotic consumption led to higher concentrations strains of *Lactobacillus* and *Bifidobacterium* [47], which are known to be reduced in the inflammatory state of microbiome dysbiosis. Given that neuroinflammation is an important factor in the pathophysiology of anxiety and depression [84], the effect of the probiotic treatment could be attributed to a reduction in serum proinflammatory cytokines such as IL-6. Similarly, in another of our studies, there was a link between fluctuations in IL-6 levels and the richness of the genus *Akkermansia*, which has been associated with proinflammatory pathways and depressive behavior in animal models [85]. Results on the BDNF changes were limited and unclear. Overall, it appears that probiotic use may be associated with a reduction in neuroinflammation and the relevant inflammatory markers. However, much more consistent data is required to reach robust conclusions.

### Limitations

Our review has a number of limitations. First, it is possible that the studies included in this review focused on patients with mild to moderate anxiety or depression, since patients with severe mental illness might be less able or willing to participate in clinical trials. In addition, some studies included in our review focused on specific patient groups (cancer, irritable bowel syndrome, insomnia), which may limit the generalizability of the results to all patients. Most studies were conducted on a single center and the data presented were sometimes based on a limited sample size. There was also a wide range in the probiotic supplements used and the duration of treatment, both of which may have influenced the results. Further, a general conclusion on whether probiotics were the main/only intervention or a complimentary one could not be reached, as there was no consistency in the additional psychopharmacological/psychological treatments participants were receiving, with a substantial number of studies not controlling for or not reporting this. Also, some studies included patients with mental disorders such as MDD or GAD, while other studies were conducted on healthy individuals with subclinical depressive or anxiety symptoms. In terms of the population studied, there was considerable heterogeneity, with some studies providing insufficient or no information on patient treatment and potential confounders such as duration of mental illness. Finally, several studies relied wholly or partly on self-reported data, whereas other studies included some form of clinical assessment.

## 5. Conclusions and Future Directions

The present comprehensive review discusses the most recent evidence from clinical trials of the last decade on the role of probiotic use in the treatment of anxiety and depression. Our findings indicate that in spite of some inconsistency in the results the majority of the recent literature appears to support the use of probiotics in alleviating depressive and anxiety symptoms. It appears that people with milder symptoms might benefit more from probiotic supplementation. In addition, a clear conclusion on probiotic-induced microbiome alterations could not be drawn, while a substantial amount of evidence seems to support a reduction in inflammatory markers associated with probiotics.

Probiotics could provide a non-drug-based approach in the treatment of mood disorders which could help expand the therapeutic options of both psychiatric and non-psychiatric patients, whilst simultaneously being safe and well tolerated. In addition, as they are nutritional supplements, probiotics could help alleviate the social stigma faced by people taking psychotropic medication. Despite advances in understanding their mechanisms of action and effectiveness, a knowledge gap still exists on where and how best to use them [41]. For instance, whether probiotics can be used as a complementary treatment combined with antidepressants or as a primary treatment for depression warrants further investigation, to evaluate their efficacy regardless of other medications. Current extensive research in the field of probiotics–mental health should focus on the exact mechanism of probiotics function, dosage, optimal treatment duration, and potential different outcomes depending on the severity of anxiety and depression. As there is still no clear evidence on which bacteria specifically contribute to the relief of depressive symptoms, future research should investigate different combinations of probiotics, as well as symbiotic and prebiotic effects alone, as their mechanisms of action might be different. Finally, from a methodological standpoint, given the challenges in analyzing highly heterogenous probiotic-related factors, such as interventions, populations, and outcome measures, future researchers should consider adhering to expert consensus methodological recommendations (e.g., on primary/secondary outcomes, definition of probiotic interventions, safety) [41].

## Figures and Tables

**Table 1:** Summary of studies’ characteristics.

Authors/Year	Type of Study	Assessment Tools for Anxiety/Depression	Findings and Comments	Probiotic Strains in the Supplement	Duration
Schaub et al., 2022 [45]	Randomized controlled trial	Hamilton Depression Rating Sale (HAM-D)—clinical assessment; Beck Depression Inventory (BDI)—self-report rating inventory; State–Trait Anxiety Inventory 1 (STAI)—self-report scale	Results show a stronger decrease in the HAM-D scores in the probiotics relative to the placebo group. The increase in the Lactobacillus was associated with decreased depressive symptoms in the probiotics group. Putamen activation in response to neutral faces was significantly decreased after the probiotic intervention.	Probiotic supplement (Isomax) containing eight different strains (*Streptococcus thermophilus* NCIMB 30438, *Bifidobacterium breve* NCIMB 30441, *Bifidobacterium longum* NCIMB 30,435 (Re-classified as *B. lactis*), *Bifidobacterium infantis* NCIMB 30,436 (Re-classified as *B. lactis*), *Lactobacillus acidophilus* NCIMB 30442, *Lactobacillus plantarum* NCIMB 30437, *Lactobacillus paracasei* NCIMB 30439, *Lactobacillus delbrueckii* subsp. Bulgaricus NCIMB 30,440 (Re-classified as *L. helveticus*)	4 weeks add-on therapy
Kazemi et al., 2019 [46]	Double-blind Randomized Controlled Trial	Beck Depression Inventory (BDI)—self-report rating inventory	Probiotic supplementation resulted in a significant decrease in BDI compared to controls and prebiotic groups.	Probiotic (*Lactobacillus helveticus* and *Bifidobacterium longum*), prebiotic (galactooligosaccharide)	8 weeks
Lee et al., 2021 [47]	Randomized, Double-Blind, Placebo-Controlled Trial	Stress Response Inventory (SRI), Beck’s Depression and Anxiety Inventory, Pittsburg Sleep Quality Index, and Insomnia Severity Index. All participants were asked to complete self-report questionnaires	The NVP-1704 group had a more significant reduction in depressive and anxiety symptoms, while also experiencing an improvement in sleep quality.	Probiotic NVP-1704, a mixture of *Lactobacillus reuteri* NK33 and *Bifidobacterium adolescentis* NK98	8 weeks
Chahwan et al., 2019 [48]	Randomised, Triple-blind, Placebo-Controlled Trial	Beck Depression Inventory (BDI)—self-report rating inventory	Probiotics did not significantly alter the microbiota of depressed individuals; however, a significant correlation was found between Ruminococcus gnavus and one depression metric.	Probiotic powder mixture: *Bifidobacterium bifidum* W23, *Bifidobacterium lactis* W51, *Bifidobacterium lactis* W52, L. acidophilus W37, *Lactobacillus brevis* W63, *Lactobacillus casei* W56, *Lactobacillus salivarius* W24, *Lactococcus lactis* W19, and *Lactococcus lactis* W58	8 weeks
Rode et al., 2022 [49]	Randomized Controlled Trial	Hospital Anxiety and Depression Scale (HAD)—self-report rating scale	Psychological symptoms, such as depression and anxiety, trended towards improvement after probiotic intervention.	Probiotic mixture containing *Bifidobacterium longum*, *Lactobacillus helveticus*, and *Lactiplantibacillus plantarum.*	4 weeks
Pinto-Sanchez et al., 2017 [50]	Randomized, double-blind, placebo-controlled study	Rome III criteria—clinical assessment; Hospital Anxiety and Depression scale (HAD)—self-report rating scale	Patients in the *Bifidobacterium longum*(BL) group had reduction in depression Scores vs. patients in the placebo group. *Bifidobacterium longum*(BL) had no significant effect on anxiety.	*Bifidobacterium longum* NCC3001 (BL)	6 weeks
Yang et al., 2016 [51]	Randomized, placebo-controlled study	Hamilton Anxiety Rating Scale (HAMA)—clinical assessment	Taking probiotics relieved the degree of anxiety of the patients. After ingestion of probiotics, serum levels of CRF did not increase before surgery.	*Clostridium butyricum*, Kexin Biotech, Shangdong, China	2 weeks
Reininghaus et al., 2020 [52]	Randomized Controlled Trial	Hamilton Rating Scale of Depression (HAMD)—clinical assessment; Beck Depression Inventory (BDI-II)—self-report rating inventory; Symptom Checklist-90-Revised (SCL-90)—clinical assessment	Probiotic plus biotin supplementation, in patient individuals with a major depressive disorder diagnosis, showed an overall beneficial effect of clinical treatment. However, probiotic intervention compared to placebo only differed in microbial diversity profile, not in clinical outcome measures.	Multistrain probiotic includes *B. bifidum* W23, *B. lactis* W51, *B. lactis* W52, *L. acidophilus* W22, L. casei W56, *L. paracasei* W20, *L. plantarum* W62, *L. salivarius* W24, and *L. lactis* W19	4 weeks
Zhu et al., 2023 [53]	Randomized, placebo-controlled study	Chinese version of Athens Insomnia Scale (AIS-8)—self-reported; Chinese version of Hamilton Depression Rating Scale (HDRS-17)—clinical assessment; Chinese version of Hamilton Anxiety Rating Scale (HAMA-14)—clinical assessment	L. plantarum JYLP-326 could be an effective strategy to alleviate anxiety and depression in test anxious college students. This effect could be related to the regulation of gut microbiota.	*Lactobacillus plantarum* JYLP-326	3 weeks
Chen et al., 2021 [54]	Preliminary Open Trial	Hamilton Depression Rating Scale (HAMD-17)—clinical assessment; Depression and Somatic symptoms Scale (DSSS)—self-report questionnaire	After PS128 intervention, scores of depression symptoms significantly decreased. However, the composition of gut microbiota did not significantly change.	*Lactobacillus plantarum* PS128	8 weeks
Tran et al., 2019 [55]	Double-Blind, Randomized Placebo-Controlled Trial	Beck anxiety inventory (BAI)—self-report rating inventory; Anxiety control questionnaire revised (ACQ-R)—clinical assessment; Positive and negative affect schedule (PANAS)—self-report questionnaire; Negative mood regulation (NMR)—self-reported; Penn state worry questionnaire (PSWQ)—self-reported	Probiotics were observed to improve panic anxiety, neurophysiological anxiety and increase negative mood regulation. In addition, participants with high distress reported higher number of improvements than those with normative distress.	Commercially available as over-the-counter products (varied)	4 weeks
Slykerman et al., 2017 [56]	Randomised Double-blind Placebo-controlled Trial	Modified versions of the Edinburgh Postnatal Depression Scale and State Trait Anxiety Inventory—self-reported questionnaires	In the postpartum phase, women who recieved HN001 scored considerably lower on depression and anxiety. The prevention or treatment of postpartum anxiety and depression symptoms may benefit from this probiotic.	*Lactobacillus rhamnosus* HN001	From enrolment until birth and, from birth up till six months post-birth whilst breastfeeding
Rudzki et al., 2019 [57]	Randomised Double-blind Placebo-controlled Trial	Hamilton Depression Rating Scale (HAM-D 17)—clinical assessment; Symptom Checklist (SCL-90)—clinical assessment; Perceived Stress Scale (PSS-10)—self-reported; Attention and Perceptivity Test (APT)—clinical assessment; Stroop Test parts A and B—both experimental and clinical purposes; Trail Making Test (TMT) Parts A and B—clinical assessment; and the California Verbal Learning Test (CVLT)—clinical assessment	Probiotic bacteria *Lactobacillus plantarum* 299v added to SSRI therapy enhanced cognitive function and reduced KYN levels in MDD patients. In comparison to the placebo group, the LP299v group’s improved cognitive capabilities may have been caused by a decrease in KYN concentration.	*Lactobacillus plantarum* 299v	8 weeks
Romijn et al., 2017 [58]	Randomised Double-blind Placebo-controlled Trial	Montgomery–Asberg Depression Rating Scale (MADRS)—clinical assessment; Improved Clinical Global Impressions scale(iCGI)—clinical assessment; Quick Inventory of Depressive Symptomatology (QIDS-SR16)—self-assessment; Global Assessment of Functioning (GAF)—clinical assessment; Depression, Anxiety, and Stress Scale (DASS 42)—self-report; Irritable Bowel Syndrome Symptom Severity Scale (IBS-SSS)—clinical assessment	Regarding all psychological outcome measures and blood-based biomarkers, there was no discernible difference between the probiotic and placebo groups. At the halfway mark, nine (23%) of the probiotic group had a change of almost 60% on the Montgomery–Åsberg Depression Rating Scale (responders), while only 10 (26%) of the participants in the placebo group exhibited the same change.	*Lactobacillus helveticus* and *Bifidobacterium longum*	8 weeks
Morales-Torres et al., 2023 [59]	Randomized Placebo-Controlled Trial	Ryff wellbeing Scale (RYFF)—self-reported; Positive and Negative Affect Scales (PANAS)—self-reported; Satisfaction with Life Scale (SWLS)—self-reported; SF-36 Health Survey; the State–Trait Anxiety Inventory (STAI)—self-reported; Difficulties in Emotion Regulation Scale (DERS)—self-reported; Multidimensional Assessment of Interoceptive Awareness (MAIA) questionnaire—self-reported; Five Facet Mindfulness Questionnaire (FFMQ)—self-reported	Findings indicated that the overall sample outcomes were not significantly impacted by probiotic consumption. Positive effects on anxiety, emotional regulation, and mindfulness in post-treatment outcomes were only predicted by the interaction between high healthy behaviors scores and probiotic intake, according to the linear mixed-effects model.	*Lactobacillus helveticus* R0052 and *Bifidobacterium longum* R0175	4 weeks
Miyaoka et al., 2018 [60]	Prospective open-label study	Diagnostic and Statistical Manual of Mental Disorders—clinical assessment; Hamilton Depression Rating Scale (HAMD-17)—clinical assessment; Beck Depression Inventory (BDI)—self-report rating inventory; Beck Anxiety Inventory (BAI)—self-report rating inventory	CBM 588 along with antidepressants provided considerable improvement in depression.	*Clostridium butyricum* MIYAIRI 588	8 weeks
Ho et al., 2021 [61]	Randomized, Double-Blind, Placebo-Controlled Pilot Trial	Beck Depression Inventory (BDI-II)—self-report rating inventory; Beck Anxiety Inventory (BAI)—self-report rating inventory; Pittsburgh Sleep Quality Index (PSQI)—self-rated questionnaire; Insomnia Severity Index (ISI)—self-report questionnaire; Epworth sleepiness Scale (ESS)—self-assessment; Heart Rate Variability (HRV); Visual Analogue Scale (VAS)—self-reported; and State Trait Anxiety Inventory (STAI)—self-reported scale	The probiotic group showed important reduces in depression scores, which are related with changes in brain waves and sleep maintenance.	*Lactobacillus plantarum* PS128	30 days
Haghighat et al., 2021 [62]	Randomized, Double-blinded, clinical trial	Hospital Anxiety and Depression Scale (HADS)—self-report rating scale	Synbiotic administration showed significant reduce in depression and anxiety symptoms and increase in the brain-derived neurotrophic factor (BDNF)	Synbiotic (15 g of prebiotics, 5 g of probiotic containing *Lactobacillus acidophilus* T16, *Bifidobacterium bifidum* BIA-6, *Bifidobacterium lactis* BIA-7, and *Bifidobacterium longum* BIA-8 (2.7 × 107 CFU/g each). Probiotics (5 g probiotics as in synbiotic group with 15 g of maltodextrin as placebo).	12 weeks
Gawlik-Kotelnicka et al., 2023 [63]	PRO-DEMET Randomized Controlled Trial	Montgomery– Asberg Depression Rating Scale (MADRS) —clinical assessment; Food Frequency Questionnaire by Wadołowska—self-reported; Depression Anxiety and Stress Scale (DASS)—self-reported; WHO Quality of Life BREF Instrument—self-report questionnaire	Probiotics have been demonstrated to be beneficial with minimal adjustment in depression cases with versus without metabolic syndrome.	*Lactobacillus helveticus* Rosell-52 and *Bifidobacterium longum* Rosell-175	60 days
Dawe et al., 2020 [64]	Randomised Controlled Trial	Edinburgh Postnatal Depression Scale (EPDS)—self-reported; State–Trait Anxiety Inventory (STAI-6)—self-report scale; 12-Item Short-Form Health Survey (SF-12-v2)—self-reported	In this diverse group of obese pregnant women, probiotics had no effect on mental health outcomes.	*Lactobacillus rhamnosus* GG and *Bifidobacterium lactis* BB12	36 weeks of pregnancy
Browne et al., 2021 [65]	Double-blind Randomized Pilot Trial	Edinburgh Postnatal Depression Scale (EPDS)—self-reported; State–Trait Anxiety Inventory (STAI)—self-report scale; MINI International Neuropsychiatric Interview	Secondary outcomes showed no significant difference between the probiotic and placebo groups on the subject of boosting maternal mood.	*Bifidobacterium bifidum* W23, *Bifdobacterium lactis* W51, *Bifidobacterium lactis* W52, *Lactobacillus acidophilus* W37, *Lactobacillus brevis* W63, *Lactobacillus casei* W56, *Lactobacillus salivarius* W24, *Lactococcus lactis* W19, and *Lactococcus lactis* W58)	26 to 30 weeks gestation until delivery
Boehme et al., 2017, [66]	Randomized, Placebo-controlled, Two-arm, Parallel, Double-blind Clinical Trial	Maastricht Acute Stress Test (MAST)—clinical assessment; Depression, Anxiety, and Stress Scale (DASS-42)—self-report; Perceived Stress Scale (PSS)—self-reported; Hospital Anxiety and Depression Scales—self-report rating scale; Gastrointestinal Symptom Rating Scale (GSRS)—self-administered; Pittsburgh Sleep Quality Index (PSQI) —self-rated questionnaire	Probiotic intervention revealed a decrease in anxiety, depression, and the cortisol awakening response.	*Bifidobacterium longum* (BL) NCC3001	6 weeks
Östlund-Lagerström et al., 2015 [67]	Double blind, Randomized, Placebo-Controlled Clinical Trial	Gastrointestinal symptoms rating scale (GSRS)—self-administered; Hospital anxiety and depression scale (HADS)—self-report rating scale; EQ-5D-5L, Perceived stress scale (PSS)—self-reported; Daily stool frequency, secondary questionnaires (e.g., HADS, PSS, EQ-5D-5L)	Neither probiotic or placebo treatment was found to be able to decrease symptoms of depression among participants suffering from gastrointestinal symptoms. Probiotic treatment has been found to have an influence on anxiety.	Freeze-dried *L. reuteri* DSM 17938, rhamnose, galactooligosaccharide, and maltodextrin	12 weeks
Lew et al., 2019 [68]	Randomised, Double-Blind, Placebo-Controlled Study	Cohen’s Perceived Stress Scale (PSS-10)—self-reported; DASS-21 stress questionnaire—self-reported; Perceived Stress Scale (PSS) questionnaire—self-reported; Depression, Anxiety, and Stress Scale (DASS-42) questionnaire—self-reported	Both placebo and P8 reduced stress levels and depression. P8 significantly exhibited lower scores as compared to the placebo for anxiety.	Probiotic *Lactobacillus plantarum* P8	12 weeks
Mohammadi et al., 2015 [69]	Randomized, Double-Blind, Placebo-Controlled Trial in petrochemical workers	General health questionnaire (GHQ)—self-administered; Depression, Anxiety, and Stress scale (DASS) scores—self-reported	Beneficial effects of probiotic supplementation may be mediated through its effects on neuronal circuits and central nervous system mediated by microbiota–gut–brain axis and the regulation of GABA receptors by the vagus nerve, known as the major regulator of the interactions between gut microbiota and the brain. Probiotics may influence both the enteric immune system by modifying the GI tract microbiome.	Probiotic yogurt contained two strains of *Lactobacillus acidophilus* LA5 and *Bifidobacterium lactis* BB12. The multispecies probiotic capsule contained seven probiotic bacteria spices *Actobacillus casei*, *L. acidophilus*, *L. rhamnosus*, *L. bulgaricus*, *Bifidobacterium breve*, *B. longum*, *S. thermophilus* and fructo-oligosaccharide with lactose as carrier substances.	6 weeks
Raygan et al., 2018 [70]	Randomized, Double-blind, Placebo-Controlled Trial	Questionnaire for Beck Depression Inventory (BDI)—self-report rating inventory; Beck Anxiety Inventory (BAI)—self-report rating scale; General Health Questionnaire-28 (GHQ-28)—self-administered	Vitamin D supplementation reduces depression score. Probiotic might improve symptoms of mental health through elevated tryptophan concentrations and reduced serotonin levels. The synergism between the immunomodulatory and anti-inflammatory effects of both vitamin D and probiotic might boost their effect on clinical symptoms of psychiatric illnesses.	Probiotic containing *Lactobacillus acidophilus*, *Bifidobacterium bifidum*, *Lactobacillus reuteri*, and *Lactobacillus fermentum*	12 weeks
Nikolova et al., 2023 [71]	Double-blind, placebo-controlled pilot randomized clinical trial	Hamilton Depression Rating Scale [HAMD-17]—clinical assessment; Inventory of Depressive Symptomatology [IDS]—self-reported; Hamilton Anxiety Rating Scale [HAMA]—clinical assessment; General Anxiety Disorder [GAD-7]—self-administered	The probiotic group attained greater improvements in both depressive symptoms scores, as well as anxiety symptoms in HAMA but not in GAD-7.	The probiotic contained 14 strains of *Bacillus subtilis*, *Bifidobacterium bifidum*, *Bifidobacterium breve*, *Bifidobacterium infantis*, *Bifidobacterium longum*, *Lactobacillus acidophilus*, *Lactobacillus delbrueckii* subsp bulgaricus, *Lactobacillus casei*, *Lactobacillus plantarum*, *Lactobacillus rhamnosus*, *Lactobacillus helveticus*, *Lactobacillus salivarius*, *Lactococcus lactis*, and *Streptococcus thermophilus*	8 weeks
Hadi et al., 2019 [72]	Randomized double-blind, placebo-controlled trial	Depression Anxiety Stress Scale-21 (DASS-21)—self-reported	Significant between-group decrease in stress, anxiety, and depression was found in the synbiotic group.	Synbiotic in form of a capsule containing *Lactobacillus acidophilus*, *Lactobacillus casei* and *Bifidobacterium bifidum* plus inulin	8 weeks
Venkataraman et al., 2021 [73]	Double-Blind, Placebo-Controlled Study	Perceived stress scale (PSS)—self-reported; Depression, Anxiety, and Stress scale (DASS)—self-reported; State–Trait Anxiety Inventory (STAI)—self-report scale	Students who consumed probiotics, showed a significant reduction in depression and anxiety scores, serum cortisol levels from the baseline as compared with placebo. No adverse effects were noted.	Multi-strain probiotic (*Bacillus coagulans* Unique IS2, *Lactobacillus rhamnosus* UBLR58, *Bifidobacterium lactis* UBBLa70, *Lactobacillus plantarum* UBLP40, *Bifidobacterium breve* UBBr01, *Bifidobacterium infantis* UBBI01 with glutamine	4 weeks
Adikari et al., 2020 [74]	Randomized, double-blinded, placebo-controlled trial	Portable electrophysiological devices	Cognitive test reaction time showed significant improvement in the probiotic group. Probiotics may offer a psychological improvement to exercise.	*Lactobacillus Casei Shirota strain*	8 weeks

## Data Availability

Data are contained within the article and Supplementary Materials.

## Supplementary Materials

The following supporting information can be downloaded at: https://www.mdpi.com/article/10.3390/microorganisms12020411/s1, Supplementary Table S1: Quality assessment of included clinical trials based on Jadad scale.
