# Supplementary material for: Probiotics’ Effects in the Treatment of Anxiety and Depression: A Comprehensive Review of 2014–2023 Clinical Trials
Source: Microorganisms. 2024 Feb 19;12(2):411. doi: 10.3390/microorganisms12020411 (PMC10893170; doi:10.3390/microorganisms12020411)
Supplement: Supplementary file 1 [file microorganisms-12-00411-s001.zip › microorganisms-2828580-supplementary.pdf]

Supplementary Table S1. Quality assessment of included clinical trials based on Jadad scale

First author, year	1. Was the study described as randomized?	2. Was the study described as double-blind?	3. Was there a description of withdrawals and drop-outs?	4. Was the randomization appropriate or inappropriate?	5. Was the blinding appropriate or inappropriate?	Quality Score
Schaub et al., 2022	YES	YES	YES	N/A	N/A	3
Kazemi et al., 2018	YES	YES	YES	YES	YES	5
Lee et al., 2021	YES	YES	YES	YES	YES	5
Chahwan et al., 2019	YES	YES	YES	YES	YES	5
Rode et al., 2022	YES	YES	YES	YES	YES	5
Pinto-Sanchez et al., 2017	YES	YES	YES	YES	YES	5
Yang et al., 2014	YES	NO	NO	YES	N/A	2
Reininghaus et al., 2020	YES	YES	YES	YES	YES	5
Zhu et al., 2023	YES	NO	NO	YES	N/A	2
Chen et al., 2021	NO	NO	YES	N/A	N/A	1
Tran et al., 2019	YES	YES	NO	N/A	N/A	2
Slykerman et al., 2017	YES	YES	YES	YES	YES	5
Rudzki et al., 2019	YES	YES	YES	YES	YES	5
Romijn et al., 2017	YES	YES	YES	YES	YES	5
Morales-Torres et al., 2023	YES	YES	YES	YES	YES	5
Miyaoka et al., 2018	YES	NO	YES	N/A	N/A	2
Ho et al., 2021	YES	YES	YES	N/A	N/A	3
Haghighat et al., 2021	YES	YES	YES	YES	YES	5
Gawlik-Kotelnicka et al., 2023	YES	YES	YES	YES	YES	5
Dawe et al., 2020	YES	YES	YES	YES	YES	5
Browne et al., 2021	YES	YES	YES	YES	YES	5
Boehme et al., 2017	YES	YES	YES	YES	N/A	4
Ostlund-Lagerstrom et al., 2015	YES	YES	YES	YES	YES	5
Lew et al., 2018	YES	YES	YES	YES	YES	5
Mohammadi et al., 2015	YES	YES	YES	YES	N/A	4

Raygan et al., 2018	YES	YES	YES	YES	N/A	4
Nikolova et al., 2023	YES	YES	YES	YES	YES	5
Hadi et al., 2019	Yes	Yes	Yes	Yes	YES	5
Venkataraman et al., 2020	YES	YES	YES	YES	YES	5
Adikari et al., 2020	YES	YES	YES	YES	YES	5
